# SAKK 24/09: safety and tolerability of bevacizumab plus paclitaxel vs. bevacizumab plus metronomic cyclophosphamide and capecitabine as first-line therapy in patients with HER2-negative advanced stage breast cancer - a multicenter, randomized phase III trial

**DOI:** 10.1186/s12885-016-2823-y

**Published:** 2016-10-10

**Authors:** Christoph Rochlitz, Martin Bigler, Roger von Moos, Jürg Bernhard, Klazien Matter-Walstra, Andreas Wicki, Khalil Zaman, Sandro Anchisi, Marc Küng, Kyung-Jae Na, Daniela Bärtschi, Markus Borner, Tamara Rordorf, Daniel Rauch, Andreas Müller, Thomas Ruhstaller, Marcus Vetter, Andreas Trojan, Ursula Hasler-Strub, Richard Cathomas, Ralph Winterhalder, on behalf of the Swiss Group for Clinical Cancer Research (SAKK)

**Affiliations:** 1 Department of Oncology, University Hospital Basel, Petersgraben 4, Basel, CH-4031 Switzerland; 2 SAKK Coordinating Center, Bern, Switzerland; 3 Department of Oncology, Kantonsspital Graubünden, Chur, Switzerland; 4 International Breast Cancer Study (IBCSG) and Inselspital, Bern University Hospital, Bern, Switzerland; 5 SAKK Coordinating Center, Bern, Switzerland and European Center for Pharmaceutical Medicine, University Basel, Basel, Switzerland; 6 Department of Oncology, University Hospital Lausanne, Lausanne, Switzerland; 7 Department of Oncology, Hospital of Valais, Sion, Switzerland; 8 Department of Oncology, Kantonsspital Fribourg, Fribourg, Switzerland; 9 Present Address: Novartis Pharma, Stein, Switzerland; 10 Department of Oncology, Spitalzentrum Biel, Biel, Switzerland; 11 Department of Oncology, University Hospital Zürich, Zürich, Switzerland; 12 Department of Oncology, Spital STS, Thun, Switzerland; 13 Department of Oncology, Kantonsspital Winterthur, Winterthur, Switzerland; 14 Department of Oncology, Kantonsspital St. Gallen, St. Gallen, Switzerland; 15 Department of Oncology, OnkoZentrum Zürich, Zürich, Switzerland; 16 Luzerner Kantonsspital, Luzerne, Switzerland

**Keywords:** Metronomic chemotherapy, Bevacizumab, Breast cancer, Toxicity

## Abstract

**Background:**

Adding bevacizumab to chemotherapy improves response rates and progression-free survival (PFS) in metastatic breast cancer (mBC). We aimed to demonstrate decreased toxicity with metronomic chemotherapy/bevacizumab compared with paclitaxel/bevacizumab.

**Methods:**

This multicenter, randomized phase III trial compared bevacizumab with either paclitaxel (arm A) or daily oral capecitabine-cyclophosphamide (arm B) as first-line treatment in patients with HER2-negative advanced breast cancer. The primary endpoint was the incidence of selected grade 3–5 adverse events (AE) including: febrile neutropenia, infection, sensory/motor neuropathy, and mucositis. Secondary endpoints included objective response rate, disease control rate, PFS, overall survival (OS), quality of life (QoL), and pharmacoeconomics. The study was registered prospectively with ClinicalTrials.gov, number NCT01131195 on May 25, 2010.

**Results:**

Between September 2010 and December 2012, 147 patients were included at 22 centers. The incidence of primary endpoint-defining AEs was similar in arm A (25 % [18/71]; 95 % CI 15–35 %) and arm B (24 % [16/68]; 95 % CI 13–34 %; *P* = 0.96). Objective response rates were 58 % (42/73; 95 % CI 0.46–0.69) and 50 % (37/74; 95 % CI 0.39–0.61) in arms A and B, respectively (*P* = 0.45). Median PFS was 10.3 months (95 % CI 8.7–11.3) in arm A and 8.5 months (95 % CI 6.5–11.9) in arm B (*P* = 0.90). Other secondary efficacy endpoints were not significantly different between study arms. The only statistically significant differences in QoL were less hair loss and less numbness in arm B. Treatment costs between the two arms were equivalent.

**Conclusion:**

This trial failed to meet its primary endpoint of a reduced rate of prespecified grade 3–5 AEs with metronomic bevacizumab, cyclophosphamide and capecitabine.

**Electronic supplementary material:**

The online version of this article (doi:10.1186/s12885-016-2823-y) contains supplementary material, which is available to authorized users.

## Background

While there is no generally accepted, optimal first-line chemotherapy regimen for mBC, most experts favor the use of taxanes and anthracyclines, either as monotherapy or in different two-drug combinations. Combination regimens typically achieve superior response rates (RR) and longer PFS than mono-chemotherapy, but have limited impact on OS [1–4] and are associated with increased toxicity [5].

The E2100 randomized phase III trial reported a near doubling of PFS and RR with paclitaxel plus bevacizumab compared with paclitaxel alone in patients with mBC [6]. Increased rates of sensory neuropathy, febrile neutropenia and infection, and severe fatigue occurred with combination therapy. These data led to the FDA (Food and drug administration) granting accelerated approval to bevacizumab plus weekly paclitaxel for the first-line treatment of HER2-negative mBC in 2008. However, this approval was later removed because: additional randomized trials [7, 8] showed less pronounced PFS and RR benefits; no trial demonstrated an OS benefit (later confirmed in meta-analyses [9, 10]); and additional safety concerns were discussed [11].

Metronomic chemotherapy is the frequent administration of chemotherapy at low, minimally-toxic doses with no prolonged drug-free intervals [12]. No trial has directly compared bevacizumab plus paclitaxel with bevacizumab plus metronomic cyclophosphamide-capecitabine. However, between-trial comparisons suggest that these regimens have similar efficacy but that toxicity with metronomic therapy is substantially reduced [6, 13]. We therefore designed a randomized phase III trial to test if metronomic chemotherapy plus bevacizumab decreases high-grade toxicity compared with paclitaxel plus bevacizumab.

## Methods

### Patients

Eligible patients had cytologically/histologically proven metastatic or locally recurrent inoperable HER2-negative breast cancer evaluable according to RECIST v1.1 criteria [14]. Other inclusion criteria included WHO (World Health Organization) performance status 0–2, low-risk for bleeding, and available baseline QoL and pharmacoeconomic assessment. Patients with (neo)adjuvant chemotherapy within the previous 6 months (12 months for taxane- and 5-FU-based chemotherapy), anti-VEGF (vascular endothelial growth factor) therapy within the previous 12 months, or prior chemotherapy for metastatic or locally advanced/recurrent breast cancer were excluded. Other exclusion criteria comprised known CNS (Central Nervous System) metastases; severe cardiovascular, renal, hepatic, or neurological disease; and history of abdominal fistula, gastrointestinal perforation, or intra-abdominal abscess.

### Trial design

This multicenter, randomized parallel open-label phase III trial compared bevacizumab plus paclitaxel versus bevacizumab plus metronomic capecitabine-cyclophosphamide as first-line therapy in patients with HER2-negative metastatic or locally recurrent breast cancer.

Patients received 10 mg/kg i.v. bevacizumab every 2 weeks with either 90 mg/m^2^ i.v. paclitaxel (days 1/8/15 of a 4 week cycle; arm A [6]) or daily oral 50 mg cyclophosphamide and 3x500 mg capecitabine (arm B [13]). All medications were given until disease progression (PD), unacceptable AE according to local investigator’s assessment, or consent withdrawal. After occurrence of an unacceptable AE to one drugs, the remaining tolerated drug(s) was (were) given until PD, consent withdrawal, or unacceptable AE.

Treatments were assigned online (www.sakk.ch/sinatras). Randomization (1:1) using minimization was stratified according to measurable/evaluable disease, WHO performance status (0/1 vs. 2), and center.

### Endpoints

The primary endpoint was the incidence of pre-specified grade 3–5 AEs (CTCAE = Common Terminology Criteria for Adverse Events, version 4.0) occurring during the trial or within 30 days of last treatment, regardless of the causal relationship to the trial drugs. These comprised: any AEs of the system/organ classes infection and infestation, febrile neutropenia, nausea, vomiting, oral mucositis, peripheral sensory neuropathy, arthralgia, myalgia, headache, thromboembolic events, cerebrovascular ischemia, left ventricular systolic dysfunction; any AEs correlated with bleeding; or any gastrointestinal perforation.

Palmar-plantar erythrodysesthesia syndrome (hand-foot syndrome, HFS), a key toxicity of capecitabine, was not a primary endpoint-defining AE in our protocol as grade 3/4 HFS was not previously observed with metronomic cyclophosphamide-capecitabine plus bevacizumab [13]. To exclude a potential bias caused by this omission we performed an exploratory sensitivity analysis that included HFS grade ≥3 as an event.

Secondary endpoints included: objective response rate, disease control rate, PFS, OS, other AEs, QoL, and pharmacoeconomics.

The primary QoL endpoint was physical well-being measured by self-assessment questionnaire at baseline (pre-randomization) and at monthly clinic visits for the first 12 months until PD. The questionnaire comprised indicators of physical well-being, mood, coping effort, overall treatment burden, health perception, appetite, tiredness, hair loss, nausea/vomiting, and numbness/tingling in hands/feet [15–20]. Higher scores (range 0–100) reflected a better condition. A change from baseline in physical well-being of ≥6 points was defined as clinically meaningful [21]. An improvement of this magnitude maintained for ≥12 consecutive weeks was defined as QoL benefit.

### Health economic analysis (HEA)

The primary endpoint of the HEA was the total incurred treatment costs until patients stopped trial treatment. The HEA adopted a health system perspective including all substantial direct medical costs incurred in the treatment of the patient. Health-related QoL was measured using EQ-5D (EuroQOL five dimensions questionnaire).

### Statistical methods

Sample size was estimated for the primary endpoint using East 5.0 and adjusted with the Casagrande-Pike-Smith correction. The expected incidence rate of predefined grade 3–5 AEs was 30 % in arm A and 10 % in arm B. With a two-sided 5 % type I error probability and 80 % power, 71 evaluable patients per arm were required. The primary endpoint was analyzed in all evaluable patients (patients who received the first bevacizumab administration of the second cycle or who experienced a primary endpoint-defining event), secondary exploratory efficacy endpoints were analyzed among all randomized patients, and secondary exploratory safety endpoints were analyzed among all randomized patients who received ≥1 dose of trial medication. All analyses were conducted using SAS 9.2 and R 3.0.0.

95 % confidence intervals were calculated for rates and arms were compared using two-sided z-tests with pooled variance and continuity correction. Time-to-event data were analyzed by Kaplan-Meier analyses and compared using log-rank tests or Gray-Tsiatis tests for cure models.

The effects of treatment allocation and time on QoL scores were estimated using mixed linear models for repeated measurements (without controlling for multiple testing).

Differences in treatment costs between arms in the HEA were tested by the Wilcoxon rank-sum test. A global multivariable linear model (proc genmod) with a gamma distribution and a logarithmic link function was used to analyze costs after controlling for age.

## Results

**Fig. 1:**
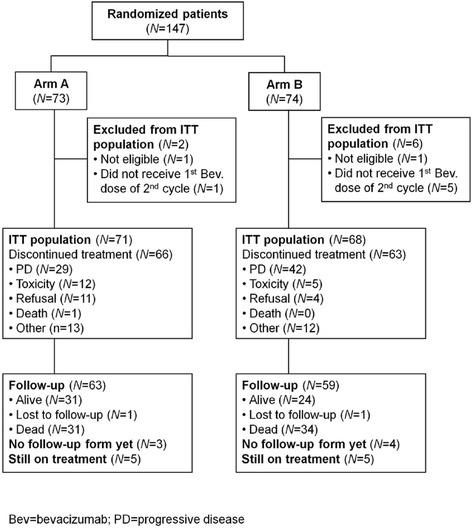
CONSORT diagram. The flow diagram shows the intention-to-treat population of 147 patients included in the primary analysis of bevacizumab plus paclitaxel (arm A) compared with bevacizumab plus metronomic chemotherapy (arm B). Bev=bevacizumab; PD=progressive disease

From September 2010 through December 2012, we randomized 147 patients to bevacizumab plus paclitaxel (*N* = 73; arm A) or metronomic therapy (*N* = 74; arm B) at 22 SAKK centers in Switzerland (Fig. 1). Patient characteristics are listed in Table 1.

**Table 1:** Characteristics of patients in the primary analysis according to chemotherapy cohort

Characteristic	Arm A (*N* = 71)	Arm B (*N* = 68)
Age – median (range)	64 (30–82)	62 (29–81)
WHO performance status, n (%)		
0, 1	66 (93.0 %)	64 (94.1 %)
2	5 (7.0 %)	4 (5.9 %)
Previous (neo-)adjuvant chemotherapy, n (%)	38 (53.5 %)	37 (54.4 %)
Previous taxane-based chemotherapy, n (%)	21 (29.6 %)	20 (29.4 %)
Estrogen-receptor status, n (%)		
negative	10 (14.1 %)	15 (22.1 %)
positive	61 (85.9 %)	52 (76.5 %)
unknown		1 (1.5 %)
Progesterone-receptor status, n (%)		
negative	23 (32.4 %)	27 (39.7 %)
positive	48 (67.6 %)	40 (58.8 %)
unknown		1 (1.5 %)
Disease evaluation, n (%)		
Evaluable	13 (18.3 %)	8 (11.8 %)
Measurable	58 (81.7 %)	60 (88.2 %)
Presence of metastases, n (%)		
Liver	41 (57.7 %)	37 (54.4 %)
Lung	25 (35.2 %)	33 (48.5 %)
Bone	52 (73.2 %)	49 (72.1 %)
Brain	0 (0 %)	0 (0 %)
Soft-tissue	12 (16.9 %)	8 (11.8 %)
Other	21 (29.6 %)	13 (19.1 %)

### Safety

The primary endpoint was evaluable in 139 patients. A primary endpoint-defining AE occurred in 18/71(25 %; 95 % CI 15–35 %) patients in arm A and 16/68 (24 %; 95 % CI 13–34 %) in arm B (*P* = 0.96). These AEs comprised neuropathy (*N* = 7), infection (*N* = 5), thromboembolic events (*N* = 3), arthralgia (*N* = 2), and nausea (*N* = 1) in arm A, and infection (*N* = 4), thromboembolic events (*N* = 4), nausea (*N* = 3), arthralgia (*N* = 2), headache (*N* = 2), and mucositis (*N* = 1) in arm B. Only one of these AEs was grade 4 (a thromboembolic event in arm A).

Results were similar in the sensitivity analysis that included HFS as a primary endpoint-defining AE: 18 (25 %) patients in arm A and 19 (28 %) in arm B had an event (*P* = 0.88).

Seventeen patients stopped treatment because of unacceptable toxicities: 12 (17 %) in arm A and five (7 %) in arm B. The main toxicities leading to suspension or delay were neuropathy, neutropenia, leukopenia, fatigue, and infection for paclitaxel; HFS, neutropenia, nausea/vomiting, and loss of appetite for capecitabine; and vomiting/nausea, thrombocytopenia, diarrhea, and neutropenia for cyclophosphamide.

### Efficacy

Objective RRs were 58 % (42/73; 95 % CI 46–69 %) and 50 % (37/74; 95 % CI 39–61 %) in arms A and B, respectively (*P* = 0.45). Four and two patients in arms A and B, respectively, achieved a complete response. Disease control rates were similar between arms (79 % [95 % CI 70–89 %] in arm A and 64 % [95 % CI 53–74 %] in arm B).

**Fig. 2:**
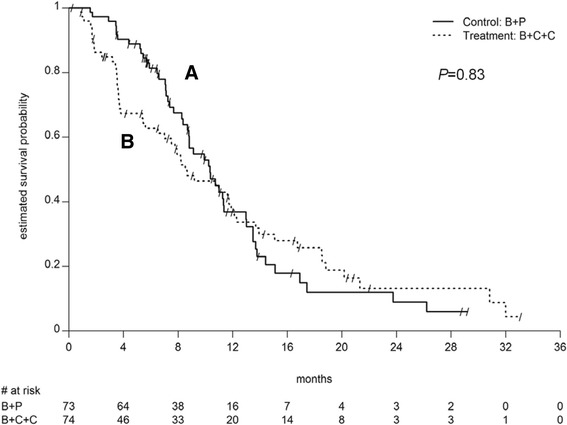
Progression free survival according to treatment arm. The *continuous line* indicates the standard arm (**a**), the *dashed line* the experimental arm (**b**)

At data cutoff, 103 patients had progressed. Median PFS did not differ between study arms (*P* = 0.83; Table 2 and Fig. 2).

**Table 2:** Progression free (PFS) and overall survival (OS)

	Arm A (*N* = 73)	Arm B (*N* = 74)
PFS		
Events, n (%)	49 (67.1 %)	54 (73.0 %)
Censored, n (%)	24 (32.9 %)	20 (27.0 %)
Median (95 % CI), months	10.3 (8.7, 11.4)	8.5 (6.5, 11.9)
Log-rank test *P*-value	0.83	
OS		
Events, n(%)	33 (45.2 %)	39 (52.7 %)
Censored, n (%)	40 (54.8 %)	35 (47.3 %)
Median (95 % CI), months	25.6 (18.9, NA)	18.7 (14.6, NA)
Log-rank test *P*-value	0.24	

*CI* confidence interval

Seventy-two patients died during a median 26.1 months follow-up (Table 2). OS was numerically higher in arm A vs arm B (*P* = 0.24).

### QoL

**Fig. 3:**
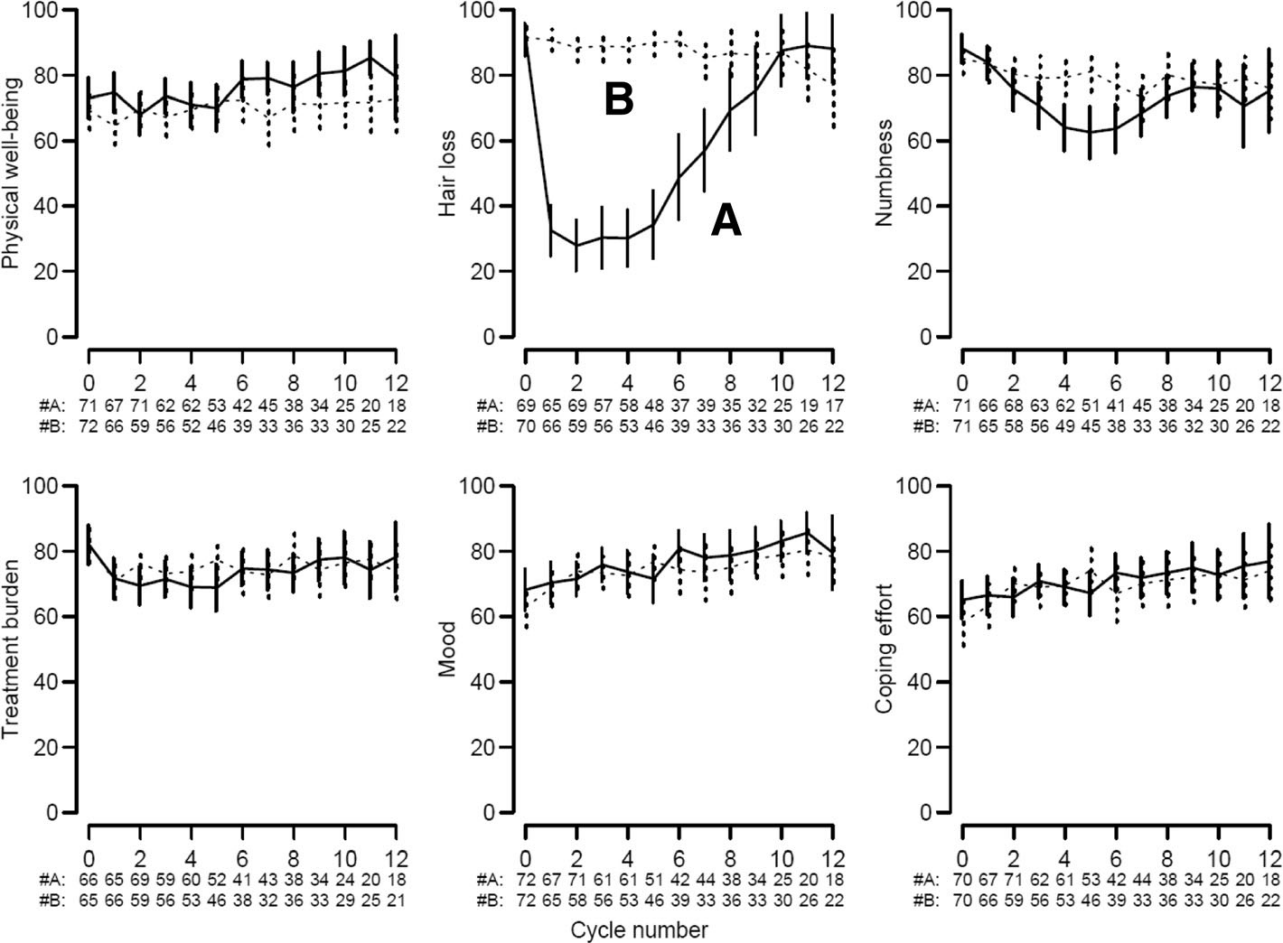
Physical well-being, hair loss, numbness, treatment burden, mood and coping effort. Data are means with 95 % CIs from baseline over 12 treatment cycles with the number of patients for each cycle. Higher scores indicate better condition. The *continuous lines* indicate the standard arm (**a**), the *dashed lines* the experimental arm (**b**)

QoL forms were available for 82 % of patients. Patients in arm B reported substantially less hair loss (*p* < 0.0001; Fig. 3) and less numbness with increasing time (*p* < 0.01) than those in arm A, and a tendency toward less overall treatment burden (*P* = 0.11). Over the first 12 months treatment, 14/70 (20 %) patients in arm A and 14/73 (19 %) in arm B indicated a QoL benefit of a similar median duration (140 and 139 days, respectively).

### HEA

By January 2014, 129 patients had stopped trial treatment (Table 3). There was no cost advantage for bevacizumab plus metronomic chemotherapy over bevacizumab plus paclitaxel. Mean total incurred treatment costs were CHF (Swiss Francs) 69,474 (~US$75,000) for arm A and CHF 80,324 (US$86,600) for arm B. Age did not significant effect the results.

**Table 3:** Health economic analysis

	Mean	95 % CI for mean	Median	SD
Arm A: Bevacizumab + paclitaxel (*N* = 66)				
TTS, months	7.3	6.3–8.2	5.9	4.0
Utility	0.8	0.7–0.8	0.8	0.2
QALM	5.9	5.0–6.8	5.1	3.7
Total incurred costs, CHF	69,474	60,624–78,324	61,815	36,001
Costs per month, CHF	10,044	9,216–10,871	9,385	3,367
Costs per QALM	14,389	11,955–16,823	11,249	9,900
Arm B: Bevacizumab + capecitabine + cyclophosphamide (*N* = 63)				
TTS, months	8.5	6.7–10.2	6.8	7.0
Utility	0.7	0.7–0.8	0.7	0.2
QALM	6.5	5.0–8.1	5.0	6.3
Total incurred costs, CHF	80,324	62,975–97,672	61,751	68,885
Costs per month, CHF	10,229	9,317–11,142	9,158	3,622
Costs per QALM	40,826	−7,257–88,909	12,025	190,922

*N* number of patients, *TTS* time to treatment stop, *QALM* quality adjusted life month, *CHF* swiss francs, *CI* confidence interval, *SD* standard deviation of the mean

Unit costs for the health economics analysis are provided in the Additional file 1: Table S1

## Discussion

We evaluated a combination of metronomic chemotherapy and bevacizumab that was described as effective and almost non-toxic in a small phase II trial [13]. We focused on grade 3/4 toxicities that are associated with a decrease in QoL and considerable additional treatment costs.

We could not demonstrate a superior toxicity profile of bevacizumab plus metronomic chemotherapy compared with bevacizumab plus paclitaxel. However, there seem to have been some qualitative differences in terms of toxicity. For instance, no grade 3/4 neuropathy was observed in the metronomic arm, whereas this adverse event occurred in almost 10 % of patients treated with paclitaxel. This toxicity is well-known to be associated with all taxanes, and especially with paclitaxel when given in a weekly regimen. Furthermore, we observed less treatment interruptions due to unacceptable toxicity in the metronomic (7 %) versus the paclitaxel arm (17 %). Although these differences were not statistically significant due to lack of power, it is highly likely that a larger study would have shown a significantly lower rate of at least some of these toxicities in the metronomic arm of the study.

The high rate of primary endpoint-defining grade 3/4 AEs in the metronomic arm of our trial is difficult to explain. These occurred in 24 % of patients in our trial but were absent in the previous phase II trial [13]. This finding might reflect differences between study populations or differences in reporting of AEs, due to the specific focus on these events in our trial. One quarter of paclitaxel-treated patients in our trial experienced primary endpoint-defining AEs, close to the incidence seen in E2100 (36 %) [6], and in more recent studies of bevacizumab in breast cancer [22]. We therefore assume that our patient population was not substantially different from that of other studies [13].

The PFS of 10.3 months observed in the taxane arm of our trial is in line with other randomized trials of first-line taxane/bevacizumab combinations (range 9.2–11.3 months) [6–8]. The PFS in the metronomic arm of our trial (8.5 months) is comparable to that reported in the phase II trial (10.5 months), though this trial also included patients in subsequent lines of therapy for advanced breast cancer [13]. Similarly, the RRs in our trial (paclitaxel arm: 58 %; metronomic arm: 50 %) are consistent with RRs in the trials upon which our study was based (E2100/RIBBON1: 37–51 %; Dellapasqua et al: 48 % [6, 8, 13]). Interestingly, a small phase II trial in 26 patients using the same metronomic therapy protocol (capecitabine, cyclophosphamide, and bevacizumab) in the same patient population (HER-2 negative mBC) as in our trial, but adding erlotinib 100 mg daily, reported a response rate of 62 % and a time to progression of 10 months compatible with but by no means proving a possible additional benefit of EGFR-inhibition in this population [23].

The QoL benefit based on patient-reported outcomes was comparable between arms. However, the reduced incidence of alopecia and numbness seen with metronomic chemotherapy might still make this regimen attractive to a substantial number of patients. The HEA failed to demonstrate a cost benefit of bevacizumab plus metronomic chemotherapy over bevacizumab plus paclitaxel. While treatment costs were not significantly different between study arms, mean (but not median) total costs were surprisingly higher when bevacizumab was combined with oral metronomic therapy versus paclitaxel. This finding is somewhat surprising on the background of previous studies and a recent review suggesting metronomic chemotherapy as a low-cost, well-tolerated, and easy to access strategy even in resource-limited countries [24].

It is tempting to speculate that both, the lack of benefit in QoL and the relatively high treatment costs in the metronomic compared to the standard arm are due to the unexpectedly high level of toxicity seen with metronomic chemotherapy in our trial.

Another possible explanation of the somewhat surprising results of our study could be subtle differences in the study populations, with for instance lung metastases being over-represented in the metronomic arm, while soft-tissue and other types of metastases appear to be more frequent in the paclitaxel arm. The randomization of patients was stratified according to measurable versus evaluable disease, performance status 0/1 versus 2, and center, which lead to a balanced distribution of these potentially prognostic/predictive factors between the two study arms. However, because of the relatively small number of cases in the trial it is impossible to determine the influence of additional factors such as the localization of metastases on our results.

## Conclusions

In conclusion, we were unable to achieve a better safety and efficacy profile for bevacizumab by combining with metronomic chemotherapy compared with paclitaxel. We therefore do not recommend further testing of this regimen of bevacizumab plus metronomic chemotherapy in mBC.

## Key message

Combining bevacizumab with metronomic cyclophosphamide-capecitabine does not achieve a superior safety and efficacy profile compared with bevacizumab plus paclitaxel in patients with advanced HER2 (human epidermal growth factor receptor 2)-negative breast cancer.

## Additional file

Additional file 1: Table S1. Unit costs for the health economics analysis. (DOCX 16 kb)

## Abbreviations

AE: Adverse events
B + C + C: Bevacizumab + cyclophosphamide + capecitabine
B + P: Bevacizumab + paclitaxel
CHF: Swiss franc
CI: Confidence interval
CNS: Central nervous system
CTCAE: Common terminology criteria for adverse events
EQ-5D: EuroQOL five dimensions questionnaire
FDA: Food and drug administration
HEA: Health economic analysis
HER2: Human epidermal growth factor receptor 2
HFS: Hand-foot syndrome
ITT: Intention to treat
mBC: Metastatic breast cancer
OS: Overall survival
PD: Progressive disease
PFS: Progression-free survival
QALM: Quality adjusted life month
QoL: Quality of life
RECIST: Response evaluation criteria in solid tumors
RR: Response rates
SAKK: Swiss group for clinical cancer research
SD: Standard deviation of the mean
TTS: Time to treatment stop
US$: US dollar
VEGF: Vascular endothelial growth factor
WHO: World Health Organization
