# Supplementary material for: SAKK 24/09: safety and tolerability of bevacizumab plus paclitaxel vs. bevacizumab plus metronomic cyclophosphamide and capecitabine as first-line therapy in patients with HER2-negative advanced stage breast cancer - a multicenter, randomized phase III trial
Source: BMC Cancer. 2016 Oct 10;16:780. doi: 10.1186/s12885-016-2823-y (PMC5057418; doi:10.1186/s12885-016-2823-y)
Supplement: Additional file 1: Table S1. — Unit costs for the health economics analysis. (DOCX 16 kb) [file 12885_2016_2823_MOESM1_ESM.docx]

**Table S1. Unit costs for the health economics analysis**

| **Resource** | **Unit costs in CHF** |
| --- | --- |
| Bevacizumab per mg | 5.55 |
| Bevacizumab application | 372 |
| Paclitaxel per mg | 1.18 |
| Cyclophosphamide per mg | 0.01174 |
| Capecitabine | 0.0095 |
| Study visit | 165 |
| CT | 633 |
| X-Ray | 140 |
| MRI | 735 |
| Ultrasound | 270 |
| PET/CT | 1500 |
| Laboratory | 150 |
| Hospital stay per day | 1600 |
| Intensive care unit per day | 2500 |
| Primary physician per visit | 90 |
| Specialist per visit | 110 |
| Hospital out-patient visit | 130 |
| Other therapies (physio-, psychotherapy, etc.) | 50–80 |
| Other medication according to Swiss tariff list^a^ | - |

^a^ http://bag.e-mediat.net/SL2007.Web.External/
